# “We will take money from anywhere to support our work”: industry funding of humanitarian assistance in crises: Jihad Makhoul

**DOI:** 10.1093/eurpub/ckac129.089

**Published:** 2022-10-25

**Authors:** J Makhoul, C El-Ashkar, R Nakkash

**Affiliations:** Health Promotion and Community Health, American University of Beirut, Beirut, Lebanon

## Abstract

**Background:**

Corporate funding has been described to be beneficial for humanitarian assistance in times of shrinking financial resources globally. Despite the growing global research on commercial determinants and their impact on population health, evaluations of corporate partnerships with humanitarian organizations and victims of multiple crises are rare. Conflicts of interest and corporate interference in public health policy and practice are well-documented. Health-harming industries are currently funding large scale projects for refugees in the eastern Mediterranean region which has witnessed humanitarian crises from armed conflicts. For example, food and beverage corporations and tobacco industries have funded projects to integrate migrants in their host countries, and offered educational scholarships to refugee children in Europe and beyond.

**Methods:**

This research presents the experiences of humanitarian agencies in Lebanon on their funding from corporations, and the perceived influences on the populations served over a two-year period coinciding with a long-lasting refugee crisis, the COVID-19 pandemic, an epic economic collapse and the devastation of a large part of Beirut from a cataclysmic explosion in its port. The study used qualitative in-depth interviews with representatives of non-governmental organizations working in Lebanon with Lebanese and refugees.

**Results:**

Funding from corporations starts with a two-way communication process between the organizations and the corporations, which recently started to be initiated by the corporations themselves after the Beirut blast. Funding from the tobacco, food and beverage industries is reported to come with conditions described to enhance their visibility, yet described as necessary, helps disadvantaged communities and sustains the organizations’ operations. Other results relating to the availability of guidelines for detecting and managing COI from corporate funding are discussed.

